# Supplementary material for: Long-term intravital imaging of the multicolor-coded tumor microenvironment during combination immunotherapy
Source: eLife. 2016 Nov 18;5:e14756. doi: 10.7554/eLife.14756 (PMC5173323; doi:10.7554/eLife.14756)
Supplement: Figure 3—source data 8. — DOI: http://dx.doi.org/10.7554/eLife.14756.023 [file elife-14756-fig3-data8.docx]

Linear fitting results_MD_Tumor periphery

MD: mean displacement

Linear fitting function: fun(ts)=a*ts+b,

where fun(ts) is the linear fitted data of MD, ts is the square root of time, a and b are fitting parameters.

gof: goodness of fit

rsquare: R^2^

“fun1, fun 2, fun 3, fun4” and “gof1, gof2, gof3, gof4” are results for Day 1, Day 3, Day 5, and Day 6, respectively..

fun1 =

General model:

fun1(ts) = a*ts+b

Coefficients (with 95% confidence bounds):

a = 7.416 (6.487, 8.344)

b = -3.999 (-5.969, - 2.029)

gof1 =

sse: 39.6612

rsquare: 0.9435

dfe: 17

adjrsquare: 0.9402

rmse: 1.5274

;

fun2 =

General model:

fun2(ts) = a*ts+b

Coefficients (with 95% confidence bounds):

a = 6.545 ( 5.92, 7.171)

b = -2.998 (-4.326, -1.671)

gof2 =

sse: 17.9979

rsquare: 0.9663

dfe: 17

adjrsquare: 0.9643

rmse: 1.0289

fun3 =

General model:

fun3(ts) = a*ts+b

Coefficients (with 95% confidence bounds):

a = 13.59 ( 12.2, 14.98)

b = - 6.72 (-9.67, -3.77)

gof3 =

sse: 88.9221

rsquare: 0.9615

dfe: 17

adjrsquare: 0.9593

rmse: 2.2871

fun4 =

General model:

fun4(ts) = a*ts+b

Coefficients (with 95% confidence bounds):

a = 15.34 (13.72, 16.96)

b = - 7.828 (-11.26, -4.394)

gof4 =

sse: 120.4788

rsquare: 0.9592

dfe: 17

adjrsquare: 0.9568

rmse: 2.6621
