# Supplementary material for: Long-term intravital imaging of the multicolor-coded tumor microenvironment during combination immunotherapy
Source: eLife. 2016 Nov 18;5:e14756. doi: 10.7554/eLife.14756 (PMC5173323; doi:10.7554/eLife.14756)
Supplement: Figure 3—source data 9. — DOI: http://dx.doi.org/10.7554/eLife.14756.024 [file elife-14756-fig3-data9.docx]

Linear fitting results_MD_Tumor parenchyma

MD: mean displacement

Linear fitting function: fun(ts)=a*ts+b,

where fun(ts) is the linear fitted data of MD, ts is the square root of time, a and b are fitting parameters.

gof: goodness of fit

rsquare: R^2^

"fun1, fun2, fun3” and “gof1, gof2, gof3” are results for Day 3, Day 5, and Day 6, respectively.

fun1 =

General model:

fun1(ts) = a*ts+b

Coefficients (with 95% confidence bounds):

a = 0.8863 (0.7753, 0.9973)

b = -0.3562 (-0.5916, -0.1208)

gof1 =

sse: 0.5663

rsquare: 0.9435

dfe: 17

adjrsquare: 0.9402

rmse: 0.1825

fun2 =

General model:

fun2(ts) = a*ts+b

Coefficients (with 95% confidence bounds):

a = 6.867 (6.226, 7.509)

b = -3.138 (-4.498, -1.778)

gof2 =

sse: 18.8952

rsquare: 0.9678

dfe: 17

adjrsquare: 0.9659

rmse: 1.0543

fun3 =

General model:

fun3(ts) = a*ts+b

Coefficients (with 95% confidence bounds):

a = 1.912 ( 1.709, 2.115)

b = -0.9353 ( -1.365, -0.5052)

gof3 =

sse: 1.8904

rsquare: 0.9588

dfe: 17

adjrsquare: 0.9564

rmse: 0.3335
