# Supplementary material for: Long-term intravital imaging of the multicolor-coded tumor microenvironment during combination immunotherapy
Source: eLife. 2016 Nov 18;5:e14756. doi: 10.7554/eLife.14756 (PMC5173323; doi:10.7554/eLife.14756)
Supplement: Figure 5—source data 7. — DOI: http://dx.doi.org/10.7554/eLife.14756.042 [file elife-14756-fig5-data7.docx]

Linear fitting results_MD

MD: mean displacement

Linear fitting function: fun(ts)=a*ts+b,

where fun(ts) is the linear fitted data of MD, ts is the square root of time, a and b are fitting parameters.

gof: goodness of fit

rsquare: R^2^

“fun1, fun2, fun3, fun4, and fun5” and “gof1, gof2, gof3, gof4, and gof5” are results for Day 0, Day 1, Day2, Day 3, and Day 4, respectively.

fun1 =

General model:

fun1(ts) = a*ts+b

Coefficients (with 95% confidence bounds):

a = 2.815 (2.66, 2.969)

b = -0.8098 (-1.138, - 0.4818)

gof1 =

sse: 1.0990

rsquare: 0.9886

dfe: 17

adjrsquare: 0.9879

rmse: 0.2543

fun2 =

General model:

fun2(ts) = a*ts+b

Coefficients (with 95% confidence bounds):

a = 6.672 (6.277, 7.068)

b = -1.66 (-2.499, -0.8207)

gof2 =

sse: 7.1906

rsquare: 0.9868

dfe: 17

adjrsquare: 0.9860

rmse: 0.6504

fun3 =

General model:

fun3(ts) = a*ts+b

Coefficients (with 95% confidence bounds):

a = 2.506 (2.253. 2.759)

b = -1.189 (-1.725, -0.6517)

gof3 =

sse: 2.9445

rsquare: 0.9625

dfe: 17

adjrsquare: 0.9603

rmse: 0.4162

fun4 =

General model:

fun4(ts) = a*ts+b

Coefficients (with 95% confidence bounds):

a = 0.7703 (0.6938, 0.8468)

b = -0.2961 (-0.4584, -0.1338)

gof4 =

sse: 0.2690

rsquare: 0.9637

dfe: 17

adjrsquare: 0.9616

rmse: 0.1258

fun5 =

General model:

fun5(ts) = a*ts+b

Coefficients (with 95% confidence bounds):

a = 1.014 (0.9396, 1.087)

b = -0.3376 (-0.5343, -0.2208 )

gof5=

sse: 0.2510

rsquare: 0.9801

dfe: 17

adjrsquare: 0.9789

rmse: 0.1215
