# Supplementary material for: Long-term intravital imaging of the multicolor-coded tumor microenvironment during combination immunotherapy
Source: eLife. 2016 Nov 18;5:e14756. doi: 10.7554/eLife.14756 (PMC5173323; doi:10.7554/eLife.14756)
Supplement: Figure 6—source data 6. — DOI: http://dx.doi.org/10.7554/eLife.14756.050 [file elife-14756-fig6-data6.docx]

Linear fitting results_MD for CTX+ACT, CTX, ACT and PBS

MD: mean displacement

Linear fitting function: fun(ts)=a*ts+b,

where fun(ts) is the linear fitted data of MD, ts is the square root of time, a and b are fitting parameters.

gof: goodness of fit

rsquare: R^2^

“fun1, fun2, fun3, fun4” and “gof1, gof2, gof3, gof4” are results for CTX+ACT, CTX, ACT and PBS, respectively.

fun1 =

General model:

fun1(ts) = a*ts+b

Coefficients (with 95% confidence bounds):

a = 6.672 (6.277, 7.068)

b = -1.66 (-2.499, -0.8207)

gof1 =

sse: 7.1906

rsquare: 0.9868

dfe: 17

adjrsquare: 0.9860

rmse: 0.6504

fun2 =

General model:

fun2(ts) = a*ts+b

Coefficients (with 95% confidence bounds):

a = 5.52 ( 5.034, 6.005)

b = -0.9605 (-1.99, 0.0695)

gof2 =

sse: 10.8383

rsquare: 0.9713

dfe: 17

adjrsquare: 0.9696

rmse: 0.7985

fun3 =

General model:

fun3(ts) = a*ts+b

Coefficients (with 95% confidence bounds):

a = 2.465 (2.287, 2.642)

b = -0.206 (-0.5817, 0.1698)

gof3 =

sse: 1.4426

rsquare: 0.9807

dfe: 17

adjrsquare: 0.9795

rmse: 0.2913

fun4 =

General model:

fun4(ts) = a*ts+b

Coefficients (with 95% confidence bounds):

a = 2.974 (2.805, 3.142)

b = -0.8559 (-1.212, -0.4993)

gof4 =

sse: 1.2993

rsquare: 0.9879

dfe: 17

adjrsquare: 0.9872

rmse: 0.2765
